# Influence of fixed titanium plate position on the effectiveness of open-door laminoplasty for cervical spondylotic myelopathy

**DOI:** 10.1186/s13018-022-03188-0

**Published:** 2022-06-03

**Authors:** Fa-jing Liu, Xiao-kun Ding, Yi Chai, Su-hong Qi, Peng-fei Li

**Affiliations:** 1 Department of Spine Surgery, Tianjin Hospital, No. 406, Jiefang Road, Hexi District, Tianjin, 300000 China; 2 Department of Internal Medicine of Traditional Chinese Medicine, Tianjin Hospital, Tianjin, 300000 China; 3 Department of Orthopedics, Hebei Provincial Hospital of Traditional Chinese Medicine, Shijiazhuang, 050013 China; 4 Department of Orthopedics, The People’s Hospital of Hengshui City, Hengshui, 053000 China

**Keywords:** Open-door laminoplasty, Cervical spondylotic myelopathy, Titanium plate, Axial symptoms, C5 palsy

## Abstract

**Background:**

During open-door laminoplasty, the position of the bone gutter is not fixed, and when the gutter migrates inward, the outer end of the titanium plate must be fixed on the lamina edge. It is unclear whether this will affect the clinical efficacy. This study aimed to observe the influence of the titanium plate fixation position on the effectiveness of open-door laminoplasty for cervical spondylotic myelopathy (CSM).

**Methods:**

A total of 98 patients with CSM who underwent open-door laminoplasty from August 2016 to October 2019 were included in this retrospective study. Fifty-five patients had the titanium plate fixed on the lateral mass (lateral mass group), and 43 patients had the titanium plate fixed on the lamina edge (lamina group). The opening angle, opening width, occurrence of hinge fracture, spinal cord drift distance, cervical curvature index (CCI), neurological function recovery (JOA score), neck function (NDI), C5 palsy and severity of axial symptoms were observed and compared between the two groups.

**Results:**

The opening angle in the lamina group was significantly larger than that in the lateral mass group, while the opening width and the spinal cord drift distance were significantly smaller than those in the lateral mass group (*P* < 0.05). The occurrence of hinge fracture in the lamina group was significantly higher than that in the lateral group (25.6% and 9.1%, respectively) (*P* < 0.05). The CCI was maintained well in both groups (*P* > 0.05), and there was no significant difference between the groups (*P* > 0.05). After surgery, the JOA score significantly increased in both groups (*P* < 0.05), and the neurological recovery rates were similar between the two groups (62.6% vs. 64.5%). The NDI score significantly decreased in both groups (*P* < 0.05), but the lateral mass group recovered to a greater degree than the lamina group (*P* < 0.05). The occurrence of C5 palsy was 2.3% in the lamina group and 14.5% in the lateral mass group, and there was a significant difference between the groups (*P* < 0.05). Postoperative axial symptom severity was significantly worse in the lamina group than in the lateral mass group (*P* < 0.05).

**Conclusions:**

In open-door laminoplasty, it is feasible to fix the titanium plate on the lateral mass or to the lamina due to the same neurological function recovery. However, fixing it to the lamina will increase the opening angle and decrease the opening width, making the hinge prone to fracture and increasing the severity of postoperative axial symptoms.

## Background

As a common spinal degenerative disease, cervical spondylotic myelopathy (CSM) occurs more often in elderly individuals and usually leads to numbness of both hands, decreased motor activity, increased muscle tension in the lower limbs, unsteady walking, and even urination and defecation functional disturbances [1]. Open-door laminoplasty is an effective surgical method for the treatment of CSM. With one side of the lamina as the axis and the other side as the door, it is opened to a certain angle to relieve spinal cord compression by increasing the volume of the spinal canal from the rear [1–3]. With the advantages of good surgical vision, simple steps, low surgical risk and simultaneous removal of multisegmental spinal cord compression, it has been widely carried out worldwide [2–4]. When a high-speed grinding drill is used to create slots on both sides of the lamina, they are often selected along the line of the transition between the laminae and the lateral masses, but there is no obvious anatomical site at this position. When patients with hyperplasia of the lamina, hypertrophy of the articular process and hypertrophy of the spinous process are encountered, the positioning of the grooves on the hinge or the open-door side may be internal or external.

Xia et al. [4] shifted the hinge gutter 2 to 3 mm inward on the laminae, which was symmetrical to the bony gutter on the open-door side, and found that this procedure could reduce the incidence of C5 palsy and the severity of axial symptoms. Lee et al. [4] believed that a position of the hinge gutter more than 1.9 mm from the pedicle on the outer cortex was a risk factor for hinge fracture. The open-door side titanium plate can be fixed to the lateral mass when the groove is opened at the transition between the lateral mass and the lamina. However, when the groove position moves inward, some laminae will remain at the inner edge of the lateral mass. At this time, the lateral end of the titanium plate can also be fixed to the lamina.

Will this fixation method affect the overall efficacy of surgery? The literature related to open-door laminoplasty has not been reported in detail. To this end, 98 CSM patients who underwent open-door laminoplasty with titanium plate fixation were included in this study. Patients were grouped according to the position of the titanium plate fixed on the open side, and then, the differences in clinical efficacy and imaging data between the two groups were observed and compared.

## Materials and methods

This retrospective study analyzed 98 patients with CSM who underwent open-door laminoplasty at Tianjin Hospital from August 2016 to October 2019. All patients were diagnosed with CSM according to their clinical manifestations and imaging data prior to surgery. According to the fixed position of the titanium plate on the open-door side, patients were divided into two groups: Fifty-five patients (31 men and 24 women) had the titanium plate fixed on the lateral mass (lateral mass group), and 43 patients (25 men and 18 women) had the titanium plate fixed on the lamina edge (lamina group). Patient data, including age, sex ratio, disease course, bone mineral density, follow-up period, and open-door segments, are shown in Table 1. Written informed consent was obtained from all patients, and this study was approved by the medical ethics committee of Tianjin Hospital.

**Table 1:** Comparison of the characteristics of the patients between the two groups

Group	*n*	Gender		Age (years)	Bone mineral density (SD)	Disease course (months)	Follow-up period (months)	Open-door segments		
		Male	Female					C3-7	C3-6	C4-7
Later mass group	55	31	24	62.5 ± 7.6	− 1.7 ± 0.6	14.6 ± 4.3	19.8 ± 5.1	37	12	6
Lamina group	43	25	18	61.9 ± 7.9	− 1.5 ± 0.4	13.5 ± 4.0	19.3 ± 5.3	30	8	5
t/χ.^2^ value		0.031		0.381	1.882	1.295	0.473	0.156		
P value		0.860		0.703	0.063	0.198	0.637	0.925		

### Inclusion and exclusion criteria

The inclusion criteria were as follows: ① typical clinical manifestations of CSM, such as numbness and weakness of limbs, holding instability, increased muscle tension, knee reflex hyperreflexia, and positive pathological signs; ② multisegmental spinal cord compression (≥ 3 segments); ③ normal cardiopulmonary, liver and kidney functions that could tolerate the surgical treatment; and ④ good compliance and could complete the postoperative follow-up on time.

Exclusion criteria: ① Combined with thoracic/lumbar spinal stenosis; ② The cervical curvature had straightened or undergone kyphosis, developmental malformation; ③ Cervical vertebral fracture, tumor, local infection and coagulation disorders.

### Surgery

**Fig. 1:**
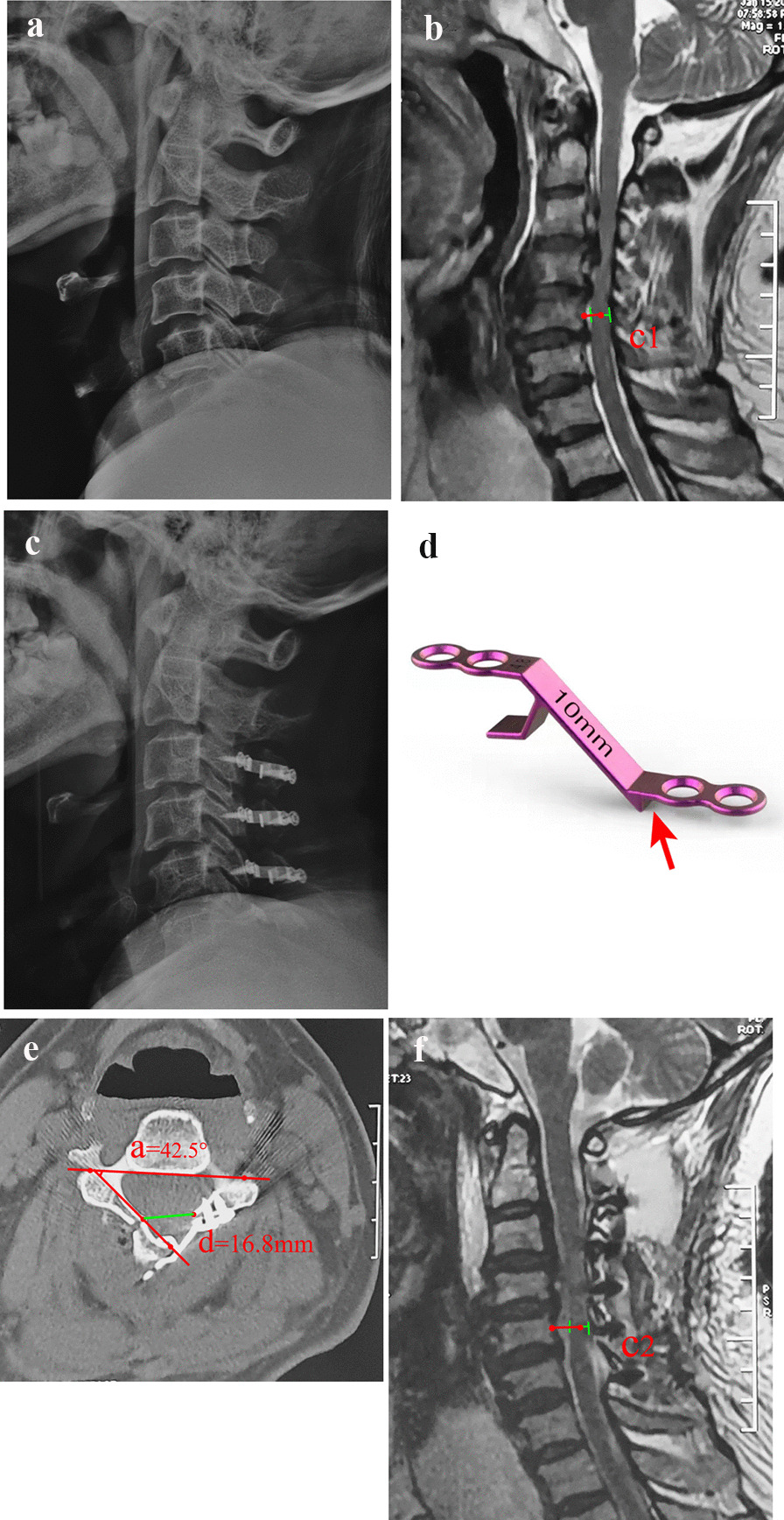
A 61-year-old male patient presented with limb numbness and intermittent claudication for 14 months and underwent open-door laminoplasty with Z-type titanium plate fixation. **a** Preoperative X-ray showing cervical degeneration and osteophytes formed at the anterior and posterior margins. **b** Sagittal MRI showing cervical disk herniations of the C_3–6_ segments. **c** Postoperative X-ray showing C_3-6_ open-door laminoplasty with titanium plate fixation. **d** Illustration of the titanium plate. The slot attached to the edge of the lamina or lateral mass (red arrow). **e** Postoperative CT showing the titanium plate fixed on the lamina edge; the opening angle was 42.5° and the opening width **d** was 16.8 mm. **f** Postoperative MRI showing that the spinal canal was enlarged with appropriate decompression and that the spinal cord drift distance was 2.4 mm

**Fig. 2:**
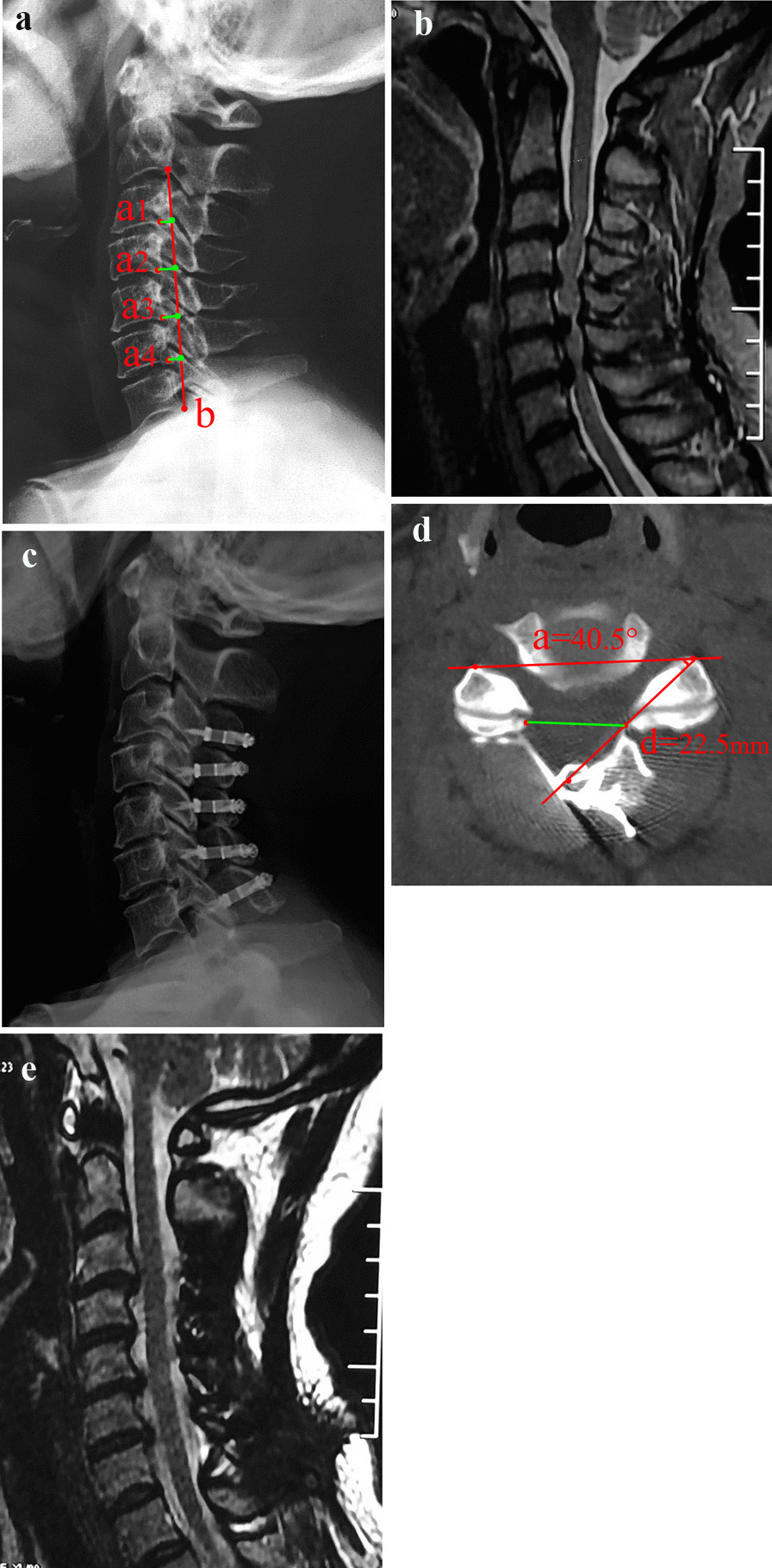
A 63-year-old male patient had typical symptoms of cervical spondylotic myelopathy for 16 months. **a** Preoperative X-ray showing the same cervical degeneration. **b** MRI showing serious spinal stenosis at the C3-7 segments. **c** Postoperative X-ray showing C_3-7_ open-door laminoplasty with titanium plate fixation. **d** Postoperative CT showing the titanium plate fixed on the lateral mass, the opening angle was 40.5° and the opening width **d** was 22.5 mm. **e** Postoperative MRI showing adequate decompression, and the spinal cord drift distance was 2.7 mm

All surgeries were performed by the same senior surgeon (Doctor Liu). After induction of general anesthesia, the patient was placed in the prone position with the head fixed in a Mayfield head frame. A posterior midline incision was made to dissect through the center of the nuchal ligament and expose the spinous processes. The posterior cervical muscles were detached from the spinous process on both sides to expose the vertebral laminae and lateral masses. A high-speed drill with a diameter of 3 mm (Stryker, USA) was used to create bilateral slots at the lamina edge or at the transition of the laminae and lateral masses. The side with less severe symptoms was used as the hinge side, and the cortex inside the vertebral lamina was retained. The side with more symptoms was used as the open side, and the medial and lateral cortices of the vertebral lamina were removed. The spinous process was trimmed, and the ligamentum flavum between the laminae was separated. After clamping the root of the spinous process, the lamina was slowly opened. An appropriate length of centerpiece titanium plate (Beijing Fule Medical Equipment Co., Ltd.) was selected and fixed on the lateral mass (lateral mass group) and laminae edge (lamina group) according to the length of the remaining vertebral laminae (Figs. 1 and 2). The wound was sutured layer-by-layer after placing the drainage tube.

The drainage tube was removed after a drainage volume < 30 ml/d. A hard collar was applied for four to six postoperative weeks.

### Evaluation criteria

The 17-point Japanese Orthopedic Association (JOA) scoring system was used to evaluate neurological recovery before and after surgery. The recovery rate was calculated as follows: (postoperative score − preoperative score) ÷ (17 − preoperative score) × 100% [5]. Axial symptoms were recorded as postoperative neck pain with neck stiffness, shoulder stiffness, or both and were evaluated by grading criteria [2, 6]: severe (analgesic or local injection regularly required), moderate (physiotherapy or therapeutic compress regularly required), or mild (no treatment required). C_5_ palsy is defined as a new occurrence of paralysis of the deltoid and/or biceps brachii after surgery, usually with mild myasthenia and C_5_ dermatome sensation disturbances [5]. The Neck Disability Index (NDI) scoring system was used to assess the neck pain and neck function preoperatively and postoperatively [3].

### Imaging evaluation

The cervical curvature index (CCI) was used to evaluate the change in cervical spine curvature. That is, on the lateral X-ray of the cervical spine in the neutral position, Line A was made from the C2 to C7 vertebral posterior inferior angle, and the vertical distance from the C3-6 vertebral posterior inferior angle to *b* was *a*1, *a*2, *a*3, and *a*4. CCI = (*a*1 + *a*2 + *a*3 + *a*4)/*b* × 100% [6]. With the help of Photoshop CS2 image measurement software (Adobe Systems Inc., United States), the opening width (d) and opening angle on the CT cross-sectional image were measured (a) (Figs. 1e and 2d). The possibility of a hinge fracture was also observed by CT scan after surgery; that is, the continuous inner cortex was terminated, and possibly, the lamina was displaced [8]. Spinal cord drift distance (C): On the midline sagittal cervical MR image at 3 months postoperatively, the distance between the posterior edge of the C5 vertebral body and the midpoint of the spinal cord was measured pre- and postoperatively, C = C2–C1 [7] (Fig. 1b and f).

### Statistical analysis

All statistical analyses were conducted using SPSS software version 22.0 (IBM, Chicago, IL, USA). Repeated measures at different time points were compared using repeated measures analysis of variance. Continuous variables were compared between the two groups using the independent two-sample t test. Proportions and grades were compared using the chi-square test and the Kruskal–Wallis rank-sum test, respectively. A two-tailed *P* value of < 0.05 was considered statistically significant.

## Results

The opening angle in the lamina group was significantly larger than that in the lateral mass group (*P* < 0.05), while the opening width and the spinal cord drift distance were significantly smaller than those in the lateral mass group (*P* < 0.05). Postoperative CT scans revealed that the occurrence of hinge fracture was 25.6% in the lamina group and 9.1% in the lateral group (*P* < 0.05). The CCI was maintained well in both groups (*P* > 0.05), and there was no significant difference between the groups (*P* > 0.05) (Table 2).

**Table 2:** Comparison of imaging data between the two groups

Group	*n*	Opening angle (°)	Opening width (mm)	Spinal cord drift distance (mm)	Hinge fracture		CCI (%)	
					Yes	No	Preop	Final follow-up
Later mass group	55	39.6 ± 5.1	21.7 ± 2.3	2.7 ± 0.5	5 (9.1%)	50(90.9%)	19.6 ± 4.5	18.7 ± 4.1
Lamina group	43	42.3 ± 5.7	17.3 ± 2.1	2.2 ± 0.4	11(25.6%)	32(74.4%)	19.2 ± 4.7	18.5 ± 3.9
t value		2.469	9.759	5.352	4.804		0.428	0.245
P value		0.015	< 0.001	< 0.001	0.028		0.669	0.807

*CCI* cervical curvature index

During the follow-up, the JOA scores increased significantly in both groups (*P* < 0.05), and there was no significant difference between the groups (*P* > 0.05). No significant difference existed in the neurological recovery rate between the two groups (62.6% vs. 64.5%) (*P* > 0.05). The NDI score significantly decreased in both groups (*P* < 0.05), but the lateral mass group recovered to a greater degree than the lamina group (*P* < 0.05) (Table 3).

**Table 3:** Comparison of neck function and neurological function recovery between the two groups

Group	*n*	NDI (%)			JOA score			Recovery rate (%)
		Preop	3 months postop	Final follow-up	Preop	3 months postop	Final follow-up	
Later mass group	55	13.6 ± 2.7	9.7 ± 1.8*	8.2 ± 1.1*	7.4 ± 1.7	12.4 ± 3.0*	13.5 ± 3.3*	64.5 ± 12.3
Lamina group	43	13.9 ± 3.0	12.6 ± 2.3*	9.4 ± 1.5*	7.2 ± 1.9	11.8 ± 2.9*	13.1 ± 3.1*	62.6 ± 11.7
t value		0.519	7.004	4.568	0.549	0.996	0.611	1.346
P value		0.604	< 0.001	< 0.001	0.583	0.321	0.542	0.181

*JOA* Japanese Orthopedic Association, *NDI* neck disability index

^*^Compared with before surgery, *P* < 0.05

The occurrence of C5 palsy was 2.3% in the lamina group and 14.5% in the lateral mass group, and the difference was significant (*P* < 0.05). There was a significant difference in the severity of axial symptoms between the two groups (*P* < 0.05), suggesting that the lamina group experienced more severe axial symptoms during follow-up (Table 4).

**Table 4:** Comparison of complications between the two groups

Group	*n*	C5 palsy		Axial symptoms		
		Yes	No	Severe	Moderate	Mild
Later mass group	55	8 (14.5%)	47 (85.5%)	2	11	42
Lamina group	43	1 (2.3%)	42 (97.7%)	4	15	24
Z/χ^2^ value		4.321		− 2.177		
*P* value		0.038		0.029		

The mean follow-up period was 19.6 ± 5.2 months. No titanium plate displacement, fracture, or laminar collapse occurred in either group.

## Discussion

In many long-term follow-up studies over the past 2 decades, open-door laminoplasty has been proven to be an effective and simple surgical procedure for the treatment of multilevel CSM [2–4, 6–8]. With the continuous development and innovation of surgical internal fixation devices, the fixation method of lamina has also changed from the initial suture suspensory to an anchor, and mini-titanium plates are widely used at this stage [3, 4, 9]. Through a clinical meta-analysis, Mo et al. [8] found that compared with suture suspensory and anchor fastening, mini-titanium plate fixation can reduce the loss of range of motion, and the incidence of axial symptoms and C5 palsy is reduced. To maintain the opening state of the lamina, the silk thread can be sutured to the opposite articular capsule, and the anchor can be placed at the midpoint of the lateral mass on the hinge side. However, the deviation of the bone gutter grinding during the operation causes the position of the hinge and the open-door to become unfixed, which further leads to it being unfixed at the lateral fixation site of the titanium plate. In the study of Xia et al. [4], the wide-open group selected the hinge trough at the inner edge of the lateral mass, while in the narrow-open group, the hinge trough was positioned at one-third outside the laminae and symmetrically to the bone gutter on the open-door side. Moving the hinge 2–3 mm inward will inevitably leave more lamina on the inner edge of the lateral mass. The residual end of the lamina will not affect the suspension and fixation of the lamina but will affect the fixation of the centerpiece titanium plate because the two ends of the titanium plate are stuck on the edge of the bone defect after the door is opened. In this study, among 98 patients who received open-door laminoplasty with titanium plate fixation, 43 patients (43.9%) had titanium plates fixed on the laminae. Will fixing the lateral end of the titanium plate on the laminae affect the clinical efficacy?

In this study, postoperative imaging measurements showed that the opening width of the lamina group was significantly smaller than that of the lateral mass group (17.3 mm vs. 21.7 mm), and the spinal cord drift distance was also smaller than that of the lateral mass group (2.2 mm vs. 2.7 mm). In addition, the opening angle of the lamina group was significantly larger than that of the lateral mass group (42.3° vs. 39.6°), but there was no significant difference in CCI between the two groups at the pre- and postoperative levels. Postoperative CT scans showed that 25.6% (11/43) of the patients in the lamina group had hinge fractures, which was significantly higher than the 9.1% (5/55) in the lateral mass group. In anatomical analysis, the inward migration of the bone gutter is bound to affect the opening width, and in the context of the same cervical curvature, the decrease in opening width will affect the distance that the spinal cord will shift backward [10]. In addition, to successfully place the titanium plate in the opening gap during the operation, it is necessary to increase the opening angle of the lamina as much as possible, and the increase in the opening angle potentially increases the risk of hinge fracture, thus triggering a series of chain reactions.

Will bone gutter inward migration affect neurological function recovery? Some studies reported [11, 12] that the transverse diameter of the spinal cord at the C5-6 segment was the widest, but only by 13 mm. Therefore, in theory, the lamina decompression width should at least exceed the transverse diameter of the corresponding segment of the spinal cord [10, 13]. Radcliff et al. [14] reviewed previous studies and found that the average laminectomy width ranged from 19 to 23.1 mm. In the study by Zhao et al. [10], the laminectomy width was controlled to 16.7 mm, and neurological function recovered significantly after the surgery. This procedure also significantly reduced the occurrence of C5 palsy. In this study, the average opening width in the lamina group was 17.3 mm, which also exceeded the maximum transverse diameter of the spinal cord. The neurological function based on JOA scores increased significantly after surgery, and there was a similar neurological recovery rate between the two groups (62.6% vs. 64.5%). C5 palsy has a striking correlation with the decompression width. The greater the width of the lamina decompression is, the more fully the dural sac bulges, and under the action of the bowstring principle, the spinal cord drift distance will increase significantly, and then, the tension on the C5 nerve root increases, leading to C5 palsy [7, 10, 13, 14]. Of course, the spinal cord drift distance is not only closely related to the width of laminectomy but also positively correlated with cervical curvature [5]. In this study, the postoperative CCI in the two groups was basically the same, so the factors affecting the spinal cord drift distance only involved the opening width. Narrowing the opening width can limit the overexpansion of the dural sac and reduce excessive backward movement under the action of the tension band. Therefore, the incidence of C5 palsy in the lamina group was significantly lower than that in the lateral mass group (2.3% vs. 14.5%).

With the widespread use of open-door laminoplasty, there are an increasing number of reports of the complications of hinge fracture [8, 15–20]. Cho et al. [16] performed CT scans on 23 patients who received open-door laminoplasty and found that 16 patients (69.6%) had one or more segment hinge fractures. In the study by Lee et al. [17], 28 (20.7%) laminae had hinge fractures in 135 opened laminae. It was previously reported that hinge fracture might be related to the thickness of the lamina [18], widths and deeps of the hinge gutter [19], opening angle [8], and hinge gutter location [20]. In this study, with the help of CT scanning, 11 patients (25.6%) in the lamina group and 5 patients (9.1%) in the lateral mass group were observed to have hinge fractures. There was a significant difference between the two groups. The total incidence was 16.3%, which was significantly lower than the rate in Cho et al. [16]. Due to steady holding by the titanium plate, the fractured hinge in both groups achieved bone union at 6 months after surgery.

In this study, the hinge gutter position in the lamina group was more inward, and correspondingly, the opening angle in the lamina group (42.3°) was larger than that in the lateral mass group (39.6°). Xu et al. [18] found that the lamina was the thickest near the spinous process and lateral mass and it gradually thinned as it approached the middle of the lamina. Therefore, the farther away the gutter position is from the lateral mass, the thinner the lamina, and the easier it is for the hinge to fracture during the opening process. At the same time, the larger the opening angle is, the greater the risk of inner cortical fracture.

Will hinge fracture affect neck function and axial symptoms? During the follow-up, the axial symptoms in the lamina group were more severe than those in the lateral mass group, and the NDI score in the lamina group was significantly higher than that in the lateral mass group at different time points after surgery. In a comparative clinical study, Wang et al. [2] found that when the lamina open-door angle was less than 40°, it could ensure adequate spinal decompression and also reduce the incidence of early and mid-term postoperative axial symptoms. Park et al. [15] believed that axial symptoms and NDI scores were closely related to hinge fracture, and patients with 3 or more segments of hinge fracture experienced significant deterioration of both axial symptom severity and NDI. Therefore, an excessive opening angle (> 40°) and hinge fracture can aggravate the severity of axial symptoms.

C5 palsy has an intimate relationship with the spinal cord drift distance because different drift distances produce different intensities of nerve root tension [10]. The lamina opening angle and opening width significantly affected the spinal cord drift distance. In our study, the spinal cord drift distance in the lateral mass group was significantly larger than that in the lamina group, and correspondingly, the occurrence of C5 palsy in the lateral mass group was significantly higher than that in the lamina group (14.5% vs. 2.3%).

## Conclusion

Compared with the method of fixing a titanium plate on the lateral mass, titanium plate fixation on the lamina edge can lead to an increase in the opening angle and a decrease in the opening width. This procedure does not affect the recovery of neurological function, but it will increase the incidence of hinge fracture and the severity of postoperative axial symptoms.

## Data Availability

The datasets generated and/or analyzed during the current study are not publicly available because the data are confidential patient data but are available from the corresponding author upon reasonable request.

## Abbreviations

CSM: Cervical spondylotic myelopathy
CCI: Cervical curvature index
NDI: Neck disability index
JOA: Japanese Orthopedic Association
